# Access to and availability of exercise facilities in Madrid: an equity perspective

**DOI:** 10.1186/s12942-019-0179-7

**Published:** 2019-07-02

**Authors:** Luis Cereijo, Pedro Gullón, Alba Cebrecos, Usama Bilal, Jose Antonio Santacruz, Hannah Badland, Manuel Franco

**Affiliations:** 1 Social and Cardiovascular Epidemiology Research Group, School of Medicine and Health Sciences, University of Alcalá, 28871 Alcalá de Henares, Madrid Spain; 2 Management and Sports Training Research Group, School of Medicine and Health Sciences, University of Alcalá, 28871 Alcalá de Henares, Madrid Spain; 3 Centre for Urban Research, RMIT University, Melbourne, Australia; 4 Urban Health Collaborative, Drexel Dornsife School of Public Health, Philadelphia, PA USA; 5 Department of Epidemiology, Johns Hopkins Bloomberg School of Public Health, Baltimore, MD USA

**Keywords:** Exercise, Socio-economic status, Exercise facilities, Inequities, Urban health

## Abstract

**Background:**

Identifying socioeconomic determinants that are associated with access to and availability of exercise facilities is fundamental to supporting physical activity engagement in urban populations, which in turn, may reduce health inequities. This study analysed the relationship between area-level socioeconomic status (SES) and access to, and availability of, exercise facilities in Madrid, Spain.

**Methods:**

Area-level SES was measured using a composite index based on seven sociodemographic indicators. Exercise facilities were geocoded using Google Maps and classified into four types: public, private, low-cost and sessional. Accessibility was operationalized as the street network distance to the nearest exercise facility from each of the 125,427 residential building entrances (i.e. portals) in Madrid. Availability was defined as the count of exercise facilities in a 1000 m street network buffer around each portal. We used a multilevel linear regression and a zero inflated Poisson regression analyses to assess the association between area-level SES and exercise facility accessibility and availability.

**Results:**

Lower SES areas had a lower average distance to the closest facility, especially for public and low-cost facilities. Higher SES areas had higher availability of exercise facilities, especially for private and seasonal facilities.

**Conclusion:**

Public and low-cost exercise facilities were more proximate in low SES areas, but the overall number of facilities was lower in these areas compared with higher SES areas. Increasing the number of exercise facilities in lower SES areas may be an intervention to improve health equity.

**Electronic supplementary material:**

The online version of this article (10.1186/s12942-019-0179-7) contains supplementary material, which is available to authorized users.

## Background

Physical inactivity and sedentarism are major health concerns, as they are estimated to cause 3.2 million deaths globally annually, predominantly through chronic diseases, especially cardiovascular diseases [1–4]. Several studies have shown a social gradient in physical activity. For example, in Spain, those with the highest level of education also have the highest physical activity levels (73.4% classified as sufficiently active), compared with those with medium or low education levels (63.1% and 38.8% classified as sufficiently active, respectively) [5]. Moreover, this social gradient represents a health equity issue in the prevalence of overweight and obesity in Madrid; residents that live in areas of lower SES have higher prevalence of obesity and overweight [6].

Population approaches [7] seek to change the distribution of risk factors within a population, through changing social determinants or environmental factors. An example of this is the neighbourhood built environment [8, 9]. Systematic variation in the characteristics of the area of residence can contribute to disparities in physical activity [10]. For instance, access to physical activity resources may vary according to the sociodemographic characteristics of the neighbourhood, such as the predominant ethnic group, the median income level, deprivation or the ageing distribution [10–14]. These may contribute to some of the differences shown between physical activity accumulation and individual-level socio-economic status [5].

Living closer to destinations that support physical activity (e.g. parks) has been associated with higher levels of physical activity [15–19]. In one hand, previous studies have showed how facility-rich environments encourage physical activity through the visual stimuli provided by the facilities presence and the numerous exercise models that they offer [20]. Secondly, it is usual that people report travel problems as a reason for quitting exercise programs [21]; living close to sport facilities reduces some psychological and physical barriers to exercise, such as travel time and traffic-related stress [20].

Evidence exists showing greater local access to sports facilities, such as gyms and sports fields, is independently associated with lower levels of adiposity [22]; other research has shown associations between the availability of exercise facilities and exercise [20]. Areas with a higher combined availability of local exercise facilities and parks have lower odds of obesity [23]. Moreover, activities supported through exercise facilities (e.g. gyms) tend to be structured and at moderate to vigorous intensity (MVPA) [24], which produces greater health benefits [25, 26]. Moreover, the structured nature of the activities of this type of facilities make this activity more related with exercise. To wit, a physical activity that is planned, structured, repetitive, and purposive in the sense that improvement or maintenance of one or more components of physical fitness is an objective [27]. Despite this, relatively little research has examined the distribution of access to and availability of exercise facilities, such as gyms or swimming pools, by SES [22].

Previous studies show a clear social gradient in the practice of physical activity [28]. In low SES areas, where crime, or perceptions of crime, is often higher [29], exercise facilities play an important role in supporting health behaviours, as the streetscape and public open spaces may not be safe and aesthetically pleasing [30]. Identifying whether there are inequities in access and availability of exercise facilities by area-level disadvantage is an important step to informing urban planning policies that can improve population health through the pathway of physical activity engagement. While some studies have looked at perceptions of exercise facility availability and its relationship with physical activity, fewer studies have used objective indicators [31, 32]. Of these studies, some lack a classification of facility types [22, 33], and those that do have a classification, have not included variables that condition access, such us price, ownership or services, but instead utilise a general typology classification [20, 34]. This is problematic because it does not allow us to know differentiated tendencies depending on the different types of facilities, specially between public and private facilities.

However, a gap in the exercise facility literature relates to the concepts of accessibility and availability. According to Penchansky and Thomas [35], accessibility incorporates the physical location of services in relation to individuals and resources required, such as transport and monetary or time costs to reach a service; meanwhile availability refers to the supply of health services, including the number and type of existing services [35]. Some studies define accessibility as the number of facilities available at a range of distances (buffers) around residents’ homes [34] or by zip code [14]; others as the number of facilities available per 1000 population [36], or whether facilities were pay- or free-for-use [13]. We argue examining accessibility and availability simultaneously provides a more nuanced understanding of the exercise facility environment for a given region. Yet, to our knowledge, no research has examined both concepts of exercise facilities within the same study.

Building on these gaps in the evidence, the aim of the study was to investigate the associations between area-level socioeconomic status with access to and availability of different types of exercise facilities and its spatial distribution using the case study of Madrid.

## Methods

### Study setting

The study is part of the Heart Healthy Hoods project, which broadly aims to study associations between the social and physical urban environment with cardiovascular health and inequity across Madrid, Spain [37].

This study was conducted across the municipality of Madrid, the capital of Spain. Madrid has a population of 3.2 M residents and is divided into 21 districts that house 128 neighbourhoods. Within each neighborhood there are small geographical administrative units of ~ 1500 people each, called census sections (N = 2415) [38]. Madrid’s socio-spatial configuration is one of the most segregated in Europe [39].

### Exposure: area-level socioeconomic status

The main exposure used in this study was a composite area-level socioeconomic status index created using seven socioeconomic status indicators: (1) low education; (2) high education; (3) part-time employment; (4) temporary employment; (5) manual occupational class; (6) average housing prices (per m^2^); and (7) unemployment rate. These indicators were selected based on the four domains present in the Spanish Commission to Reduce Health Inequalities [40] (education, wealth, occupation and living conditions). Occupation and living conditions indicators were assessed at the neighbourhood level. The area-level disadvantage index was calculated for each census section of the study area. The index has been used in other research [41], and further details regarding index construction are described in Additional file 1. For the purposes of this paper, the index was collapsed into deciles, where 1 = most disadvantaged census sections and 10 = least disadvantaged census sections.

### Outcomes: exercise facilities

Exercise facilities were defined as indoor exercise facilities, both public and private, which offered physical activity programs, both with monthly subscription or pay per session (e.g. fitness clubs, sport centres, dance clubs, Pilates studios). Informal facilities (e.g. public parks or outdoor playing fields), cycling paths, private clubs (e.g. exercise facilities not accessible to the public, schools, or private sport clubs) were excluded.

Exercise facility information was collected by *‘MAS Servicios Integrales’* between April and October of 2015. All exercise facilities across Madrid were identified by Google Maps. Information about the programs and services were sourced through telephone and face-to-face interviews with facility managers. All facilities were visited physically to check the information collected. Data collection was carried out by four trained observers. Quality assurance was carried out by repeating the above process again in two districts using different trained data collectors.

The database used in this study comprised of 595 exercise facilities with five variables on facility characteristics. These were: (1) Name of the facility; (2) Address; (3) Monthly price; (4) Type of sports programs and services offered; (5) Ownership (public vs private). The exercise facilities were further classified into four exercise facility ‘types’, as described in Table 1. Similar classifications have been used in previous studies [14, 34].

**Table 1:** Descriptive analysis of the exercise facilities about accessibility and availability

Exercise facility type	Definition	*N*	Accessibility			Availability		
			Median (m)	IQR		Median (count)	IQR	
All the facilities		595	369.89	222.94	603.89	5	2	9
Publicly owned	Monthly payment option. Public ownership	59	1058.35	713.39	1466.25	0	0	1
Privately owned	Monthly payment ≥ 30€/month. Private ownership	222	611.42	353.53	1042.11	2	0	4
Low cost	Monthly payment < 30€/month. Private ownership	63	1092.23	666.42	1791.08	0	0	1
Sessional	Facilities with Pay-per-session (e.g. Pilates Studios, Dance Schools, electrostimulation centres…). Private ownership	251	594.35	328.49	1036.33	2	0	4

*IQR* interquartile range; *m* meters

### Portal

We identified all residential building entrances in the city from CARTOCIUDAD [42] by identifying all external access identifiers located in a residential land use (total n of 125,440. We exclude entrances whose nearest facility was located more than 6 km away (N = 13), as these entrances were located in the edge of the city of Madrid, and their closest exercise facility might not be in the city in Madrid, but in a surrounding small region. All the spatial measures were calculated using ArcGIS 10.1 software.

### Measure of accessibility to exercise facilities

We calculated the distance from each portal (origin) to the nearest exercise facility (destination) using a street network analysis; this better represents the true spatial distance between points when compared with a Euclidean distance [43]. We calculated the distance to “any” exercise facility less than 6 km, and the distance to the nearest facility of each type (Table 1).

### Measure of availability of exercise facilities

We calculated the availability (count) of exercise facilities in total and by type using a 1000 m street network buffer. There is empirical evidence suggesting 1000 m is the distance people are most likely to walk to fulfil daily activities [43]. In fact, previous studies showed that 1000 m from home to an exercise facility is the distance with the highest correlation with moderate to vigorous physical activity [44], and this distance has previously been applied in exercise facility research [22, 44, 45].

### Mapping of spatial distribution

Two cartographic maps were developed to facilitate the visualization of the spatial distribution of exercise facilities in terms of accessibility and availability. Those maps were made from the calculation of the average distance to the nearest exercise facility (accessibility) and number of exercise facilities 1000 m around (availability) of each census section.

### Statistical analyses

To study the association between accessibility to the nearest exercise facility and area-level SES we used linear mixed models with log transformed distance as the dependent variable and the SES index as the independent variable. This was a three-level model with a random intercept for neighbourhood and for census section. We included the independent variable (SES Index) operationalized as deciles, with the first decile (lowest SES) as the reference, group. To study the relationship between availability of exercise facilities and area-level SES, we used a Zero Inflated Poisson (ZIP) model. We chose a ZIP model instead of a mixed effects Poisson due to the high number of 0’s in the distribution of the dependent variable. We estimated robust standard errors clustered by census section to take into account the intra-census section correlation. We ran all models for all facilities and stratified by type of facility. All analyses were conducted using Stata/SE 14.1 for Mac (StataCorp., College Station, TX, USA).

## Results

Overall, the median distance to the nearest exercise facility (any type) from each portal was 364 m (IQR = 220 m–596 m). By type, low-cost facilities were furthest away (median distance = 1090 m, IQR = 663 m–1789 m), and sessional facilities were most proximate (median distance = 596 m, IQR = 331 m; 1035 m) (Table 1).

All portals had two or more exercise facilities of any type located within 1000 m, and half of the portals had at least five facilities available at this distance. However, half of the portals had neither public exercise facilities nor low cost facilities available within 1000 m. Private and sessional facilities had the highest availability, with at least two exercise facilities available within 1000 m for half the portals.

### Exercise facility accessibility and SES

**Fig. 1:**
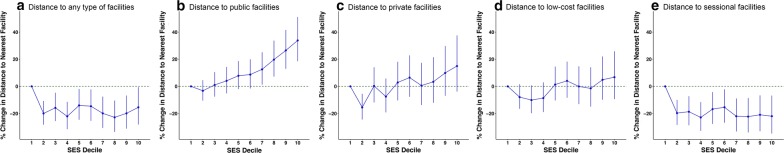
Area-level SES and accessibility to nearest exercise facility. *Note* Distance = logarithm of distance to nearest facility; SES = socio-economic status

Overall, there was a social gradient in public, private and sessional facilities, where portals in low SES areas have better accessibility to the nearest exercise facility compared with higher SES areas (Fig. 1b–d). However, this association differed by type of facility. Areas with lower SES had higher accessibility to public exercise facilities (Fig. 1b). Similar patterns, though less strong, were observed for privately owned facilities (Fig. 1c) and low-cost facilities (Fig. 1d). In the case of sessional facilities, this gradient was unclear. Despite this, portals in the lowest SES areas (decile 1) had the lowest accessibility to the nearest exercise facility. This was shown for all types of exercise facilities when compared with the next least-deprived SES decile.

**Fig. 2:**
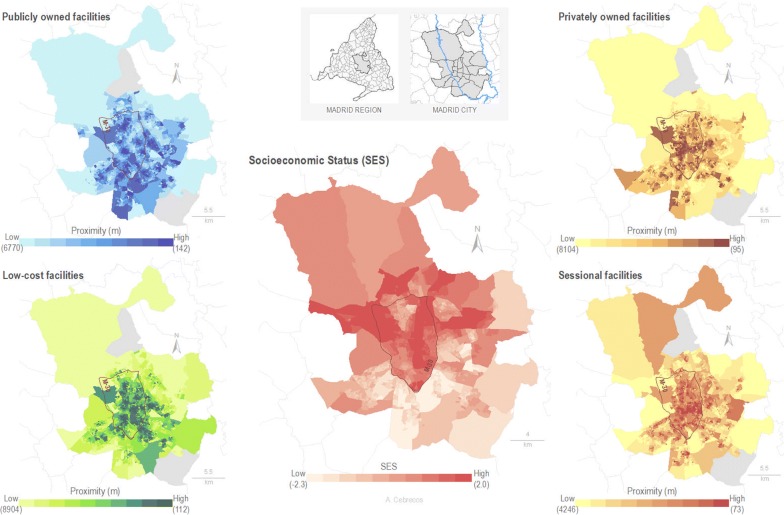
Spatial distribution of census section average distance from each portals to the nearest exercise facilities by type, and Area-Level Socio-Economic Status Index by deciles in the census section (N = 2415) of the city of Madrid. *Note* m = meters; SES = socio-economic status

The spatial distribution of area-level SES and average distance to the nearest exercise facilities by type is shown in Fig. 2. The portals of the down-town area of Madrid (inside the M-30 orbital motorway of Madrid) show shorter distances to exercise facilities. Public exercise facilities are more accessible in the southern areas of the city when compared with the north, meanwhile the sessional exercise facilities show the opposite relationship. Low-cost and private exercise facilities were located most proximally in the downtown and southeastern areas. Private exercise facilities were located most proximally in the southwestern region.

### Exercise facility availability and SES

**Fig. 3:**
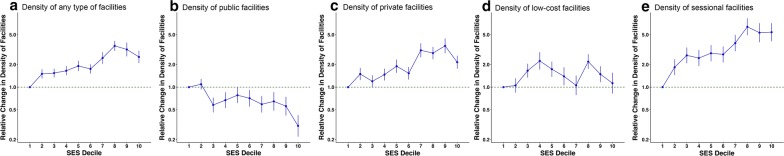
Area-level SES and availability of exercise facilities. *Note* IRR = incidence rate ratio; SES = socio-economic status

There is a reverse social gradient in the association to availability of exercise facilities (Fig. 3), as there is a higher density of facilities in higher SES areas. The strongest associations with availability were shown for private and sessional exercise facilities, with the number of facilities increasing in areas of higher SES. This patterning was not present when public or low cost exercise facilities were considered.

**Fig. 4:**
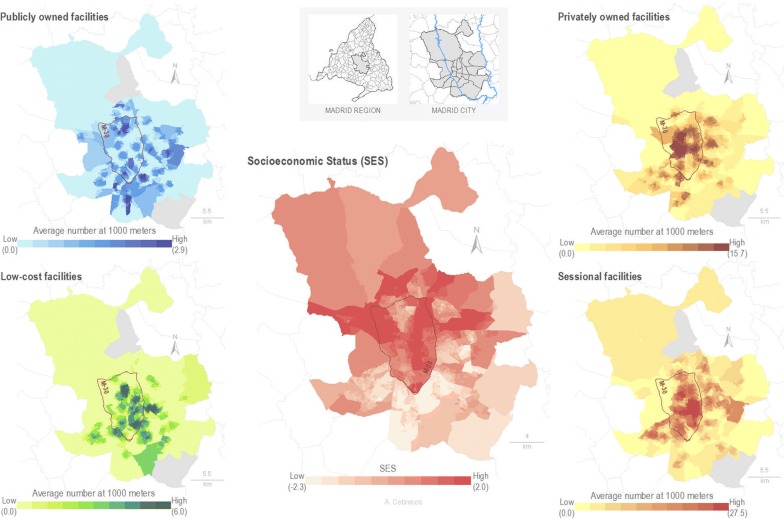
Spatial distribution of census section average availability from each portal to exercise facilities by type using a 1000 m street network buffer, and Area-Level Socio-Economic Status Index by deciles in the census section (N = 2415) of the city of Madrid. *Note* SES = socio-economic status

Figure 4 shows differences in the spatial distribution of the availability of exercise facility types between the down-town area of Madrid and the periphery of the city. Downtown and northern areas (high SES) have greater availability of all types of exercise facilities. Public facilities have a higher level of availability when compared with other facility types, especially in the southern part of the city. Private and low-cost facilities have higher availability in the lower SES areas of the south than sessional facilities, which are more present in the higher SES areas of the north.

## Discussion

This study showed that, while people in Madrid living in low SES areas had better access to exercise facilities, residents in higher SES areas had greater availability of exercise facilities. The relationship between accessibility and area-level SES varied depending on the type of exercise facility, yet all types (except sessional facilities) presented a social gradient where distances generally tended to be more proximal in low SES areas. This gradient was most strong for the publicly owned and low-cost facilities. The availability analysis showed an increased likelihood of having more than one facility available as area-level SES increased. This pattern was clearest with private and sessional facility types. Our paper builds on this novelty research by further classifying exercise facilities into types, collecting primary data, and examining the socio-spatial patterning of exercise facilities by access and availability.

Our results are consistent with previous research that showed a negative association between area-level SES and proximity, in terms of distance, to recreational facilities [46–48], green spaces [47, 49, 50], and playgrounds [51, 52]. However, other studies have shown a greater proximity of green spaces for high SES areas, but not for other facilities [53–55]. This suggests that exercise facilities accessibility could act as a barrier of social disadvantaged, as an “advantage in the disadvantaged [41, 56].

Our availability results are consistent with previous studies that demonstrated higher density of facilities in areas of higher SES [13, 14, 34, 57]. Other studies, developed in different countries, found that there are more facilities in lower-SES areas [58, 59], while others have reported mixed or null results [60]. A previous study carried out in Madrid with older adults and secondary data sources showed similar availability of exercise facilities to those of our study. It concluded that reduced availability of exercise facilities in disadvantaged populations was a contributor to physical inactivity in older adults [36].

While the low SES areas had better accessibility and lower availability of exercise facilities, the higher SES areas presented opposite relationships. This could be explained by a high concentration of exercise facilities in the centre of Madrid, where census sections tended to have higher SES; on the other hand, the neighbourhoods on the periphery of the city (lower SES, especially in the south) have a more dispersed distribution of exercise facilities.

### Strengths and limitations

This is the first multilevel study that: (1) analysed exercise facilities, in terms of accessibility and availability, and examined this in relation to area-level disadvantage; and (2) classified and examined exercise facilities based on price, subscription type and ownership.

The results show the importance of doing research that incorporates both access and availability measures simultaneously, and when combined with SES, can reveal different (and sometimes opposite) social-spatial patterning and social gradients. Examining the different types of exercise facilities yielded diverse results when considered by area-level SES, particularly in relation to generating a better understanding of the (in)equities of delivery. Another strength was the use of primary data for exercise facilities and accessibility and availability measures in Madrid. Finally, using the whole municipality of Madrid provided a high level of population variation to examine the socio-spatial distribution of exercise facilities.

Some limitations of this study should be highlighted. This research did not take into account the impact of the accessibility and availability of exercise facilities with behaviours of the population, such as facility use or physical activity engagement. There have also been concerns that area-level SES measures may not be suitable proxies for individual-level SES because of potential disagreement between contextual and compositional effects [61]. Because of the absence of individual data, portals were used to estimate accessibility and availability of exercise facilities from the residences of the Madrid population; however, exercise facilities around workplaces and/or study centers, may also be important but were not investigated. Finally, our focus was exercise facilities, therefore, we might have missed other physical activity destinations, such as playgrounds or parks. However, we chose to restrict to study exercise facilities since the activities supported in exercise facilities (e.g. gyms) tend to be more structured and include moderate to vigorous intensities (MVPA) [24], which produces greater health benefits [25, 26].

### Policy recommendations

Presence of exercise facilities have a great importance on the physical activity engagement of the populations. Not only for the type of the structured activities provided [24], but also for the impact on the neighbourhood environment [20].

In one hand, previous studies have pointed how the facility-rich environment encourage physical activity through the visual stimuli provided by the own facilities and the numerous role models presence thanks to the nearby facilities [20]. Secondly, is usual that people report inconvenience and travel problems as reasons for quitting of exercise programs [21]; to live near facilities reduce some psychological and physical barriers to exercise, such as travel time and traffic-related stress [20].

Previous studies have reported a positive relationship between the availability of exercise facilities and moderate to vigorous physical activity [62] and a negative relationship with adiposity [22]. Therefore, the low availability of exercise facilities detected in areas with low SES brings a double disadvantaged scenario for those populations, such as in the southern districts of Villaverde and Puente de Vallecas, as well as some areas in the southeast part of the city.

An increase of opportunities for physical activity in more disadvantaged areas, either through subsidy systems of private facilities or increasing the availability of public facilities, could produce an upturn in the aggregate demand of physical activity. This planned growth should focus on low fixed price or no cost facilities, as those with a variable price (such as sessional) are negatively related to participation in physical activity [63], and may be a barrier for those who are disadvantaged people.

### Research agenda

Future studies should try to extend our findings using individual-level behavioural data to better understand how exercise environment is associated with facility use and physical activity engagement. In future, a wider range of internal characteristics of the facilities should be assessed (e.g. service quality, cultural appropriateness, timetabling), alongside understanding how these attributes are associated with facility use. Also, a qualitative approach to evaluate the characteristics of exercise facilities could improve our understanding of the barriers/enablers people face when selecting (or not) exercise facilities to attend, and whether this differs by SES.

## Conclusions

Our findings showed that associations between accessibility and availability of exercise facilities with area-level SES varied depending on facility type. Areas with lower SES demonstrated better accessibility in general to exercise facilities, whereas higher SES areas had greater facility availability, especially when privates and sessional types were considered.

Relatively little research to date has examined exercise facilities, when compared with evidence focussing on other physical activity locations, such us parks or neighbourhoods. This study makes an important contribution to knowledge about the socio-spatial delivery of exercise facilities in our cities.

## Additional file

**Additional file 1.** Area Level Socioeconomic status indicators.

## Data Availability

The data that support the findings of this study are available from MAS Servicios Integrales but restrictions apply to the availability of these data, which were used under license for the current study, and so are not publicly available. Data are however available from the authors upon reasonable request and with permission of MAS Servicios Integrales.

## Abbreviations

SES: socio-economic status
MVPA: moderate to vigorous physical activity
