# Supplementary material for: Access to and availability of exercise facilities in Madrid: an equity perspective
Source: Int J Health Geogr. 2019 Jul 2;18:15. doi: 10.1186/s12942-019-0179-7 (PMC6604462; doi:10.1186/s12942-019-0179-7)
Supplement: Supplementary file 1 — Additional file 1. Area Level Socioeconomic status indicators. [file 12942_2019_179_MOESM1_ESM.docx]

**Additional file 1.** Area Level Socioeconomic status indicators.

| **Construct** | **Domain** | **Indicator** | **Operationalization** | **Source** | **Level** |
| --- | --- | --- | --- | --- | --- |
| SES | Education | Low Education | Residents with mandatory studies or below / all residents aged 25 years or above | Padron | Census Section |
|  |  | High Education | Residents with university education or above / all residents aged 25 years or above | Padron | Census Section |
|  | Occupation | Part time Jobs | Workers in part-time jobs / all workers | Social Security | Neighbourhood |
|  |  | Temporary Jobs | Workers in temporal jobs / all workers |  |  |
|  |  | Manual Occupation Class | Workers in manual or unskilled occupations / all workers |  |  |
|  | Wealth | Housing Prices | Average sale price of housing per m^2^ | Idealista Report | Census Section |
|  | Living Conditions | Unemployment Rate | Residents registered as unemployed / all residents aged 16–64 years | Employment Service | Neighbourhood |

**Key:** SES= Socio-Economic Status
